# Neobavaisoflavone May Modulate the Activity of Topoisomerase Inhibitors towards U-87 MG Cells: An In Vitro Study

**DOI:** 10.3390/molecules26154516

**Published:** 2021-07-27

**Authors:** Mateusz Maszczyk, Zuzanna Rzepka, Jakub Rok, Artur Beberok, Dorota Wrześniok

**Affiliations:** Department of Pharmaceutical Chemistry, Faculty of Pharmaceutical Sciences in Sosnowiec, Medical University of Silesia in Katowice, Jagiellońska 4, 41-200 Sosnowiec, Poland; d200888@365.sum.edu.pl (M.M.); zrzepka@sum.edu.pl (Z.R.); jrok@sum.edu.pl (J.R.); abeberok@sum.edu.pl (A.B.)

**Keywords:** neobavaisoflavone, glioblastoma, doxorubicin, temozolomide, irinotecan, etoposide

## Abstract

Despite many advances in therapy, glioblastoma (GB) is still characterized by its poor prognosis. The main reason for this is unsuccessful treatment, which slightly extends the duration of remission; thus, new regimens are needed. One of many types of chemotherapeutics that are being investigated in this field is topoisomerase inhibitors, mainly in combination therapy with other drugs. On the other hand, the search for new anti-cancer substances continues. Neobavaisoflavone (NBIF) is a natural compound isolated from *Psoralea corylifolia* L., which possesses anti-oxidant, anti-inflammatory, and anti-cancer properties. The aim of this study was to evaluate the effect of NBIF in human U-87 MG glioblastoma cells in comparison to normal human NHA astrocytes, and to examine if it influences the activity of irinotecan, etoposide, and doxorubicin in this in vitro model. We demonstrated that NBIF decreases U-87 MG cells viability in a dose-dependent manner. Furthermore, we found that it inhibits cell growth and causes glutathione (GSH) depletion more intensely in U-87 MG cells than in astrocytes. This study also provides, for the first time, evidence of the potentialization of the doxorubicin effect by NBIF, which was shown by the reduction in the viability in U-87 MG cells.

## 1. Introduction

Glioblastoma (GB), a grade IV glioma, is the most frequent primary tumor located in the central nervous system. It is also one of the deadliest cancers [1,2]. Approximately only 5% of patients diagnosed with this neoplasm survive 5 years after the recognition of the disease [3]. The main reasons for such low survival are late diagnosis and ineffective treatment connected with such difficulties as drug resistance and tumor heterogeneity. At present, the standard for GB treatment concerns surgical removal of the tumor [4]. Due to the deep infiltration of GB cells into brain tissue, postoperative patients undergo radio- and chemotherapy. However, the current therapeutic strategy slightly extends the duration of remission, and the tumor typically recurs [5,6]. 

The major pharmaceuticals used in GB treatment are alkylating agents, such as temozolomide (TMZ) [7]. Its mechanism of action involves methylation of guanine at the O6 position, which results in DNA damage and apoptosis of GB cells [8]. Nonetheless, clinical data indicate that TMZ is often inefficient [5,8]. This is due to the activity of O6-methylguanine-DNA methyltransferase (MGMT), a DNA repair enzyme, which limits the effect of TMZ [2,5,8]. The expression of its gene is elevated in up to 60% of GB cases, making it a major obstacle to successful treatment [9]. Thus, there is a strong need for a new therapeutic approach.

A group of the anti-cancer agents that might be applied in GB therapy is topoisomerase inhibitors, such as irinotecan, etoposide, or doxorubicin. They are widely used in the treatment of many types of cancer, e.g., breast, colorectal, lung, gastric, and ovarian [10,11]. Numerous clinical studies have shown that they also have anti-cancer potential against GB, mainly merged with other chemotherapeutics [11,12,13,14,15,16]. For instance, irinotecan combined with bevacizumab, an anti-angiogenic humanized monoclonal antibody inhibiting activity of the vascular endothelial growth factor (VEGF), was proved to be effective in patients with recurrent GB [12,15]. A similar point was made for the combination chemotherapy regimen of etoposide and bevacizumab [13,14]. Doxorubicin alone or merged with TMZ was shown to induce cell death in GB cells in pre-clinical studies [11,16] and extend survival in the in vivo GB models [17,18]. However, due to the limitations in blood–brain barrier permeability of doxorubicin, and other topoisomerase inhibitors as well, adequate methods of drug delivery should be considered [19]. Major advances in this field may be a chance for their implementation in GB treatment in the future.

In recent decades, substances of natural origin have been in the spotlight of researchers worldwide. A large group of chemicals obtained from plants is polyphenols, which are defined as compounds containing one or more aromatic rings with at least one hydroxyl group attached [20]. They are divided into five main subgroups: flavonoids, lignans, phenolic acids, stilbenes, and other polyphenols [20,21]. A continuously growing body of evidence indicates that these compounds have advantageous effects on human health and might be potent in the prevention and therapy of many diseases, such as diabetes, cardiovascular illnesses, neurogenerative disorders, and cancer [21,22]. The properties of polyphenols derive from their anti-oxidative and anti-inflammatory activities, which protect normal cells from damage, thus preventing the development of the disease [22]. Polyphenols are also able to affect abnormal cells through the modulation of the signaling pathways involved in proliferation, migration, angiogenesis, and apoptosis [20]. Molecular mechanisms underlying their action are diverse and complex. Among others, polyphenols cause inhibition of transcription factors associated with these processes, such as nuclear factor NF-κB. Its main function concerns an activation of inflammation and can be triggered by such stimuli as oxidative stress. Downregulation of NF-κB leads to the reduction in cell proliferation and the induction of cell death [23]. Polyphenols can also cause these effects through the inhibition of signaling pathways crucial for cell survival and growth, such as phosphatidylinositol-3-kinase (PI3K)/protein kinase B (AKT) and mitogen-activated protein kinase (MAPK)/extracellular regulated kinase (ERK) pathways, and also by downregulation of the anti-apoptotic proteins, such as Bcl-2 [24].

Neobavaisoflavone (NBIF), an isoflavone found in *Psoralea corylifolia* L., possesses numerous beneficial health properties, including anti-inflammatory, anti-oxidative, and osteogenic effects [25,26]. It has also been claimed that NBIF has anti-cancer properties [27]. NBIF was proved not only to decrease viability in several different cancer cell lines but also to enhance apoptosis in cells resistant to tumor necrosis factor-related apoptosis-inducing ligand (TRAIL) [27,28,29]. Thus, NBIF shows potential as a chemopreventive agent and needs to be investigated further. This compound was also claimed to have neuroprotective effects [30]. Therefore, it might be studied in the field of brain malignancies. Additionally, the evidence of isoflavones improving the efficacy of cancer chemotherapy suggests that NBIF also might influence the activity of some drugs [31]. 

The aim of the present study was to evaluate the effect of NBIF on cell proliferation and homeostasis in human U-87 MG glioblastoma cells in comparison to normal human NHA astrocytes, and to examine its influence on the activity of irinotecan, etoposide, and doxorubicin in this in vitro model. To meet this goal, we analyzed the impact of NBIF on cell viability and growth of U-87 MG and NHA cells. We also determined its influence on the intracellular GSH level, which reflects cellular homeostasis. Based on the results obtained, in the next part of the study, we performed a WST-1 assay to investigate how NBIF affects the activity of irinotecan, etoposide, doxorubicin, and additionally, TMZ on GB cells.

## 2. Results

### 2.1. NBIF Decreases Viability and Amount of NHA and U-87 MG Cells without Affecting the Cell Cycle

**Figure 1:**
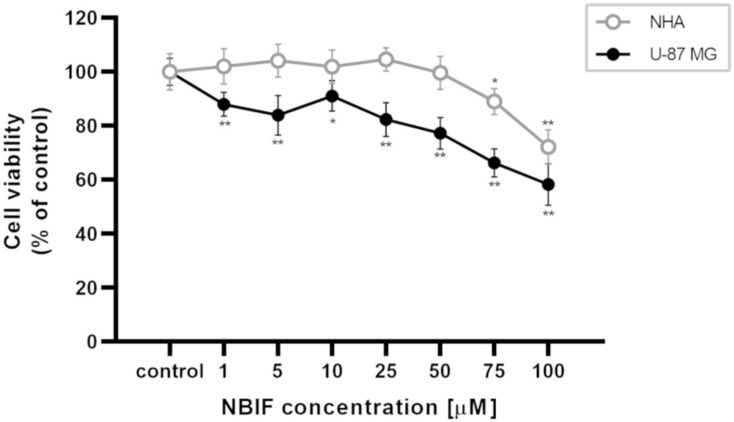
The effect of neobavaisoflavone (NBIF) on viability of NHA human astrocytes and U-87 MG glioblastoma cells. Cells were treated with neobavaisoflavone concentrations ranging from 1 μM to 100 μM and control for 48 h and examined by the WST-1 assay. Mean values ± SD of three independent experiments are presented. * *p* < 0.05, ** *p* < 0.001 vs. control.

To determinate the effect of NBIF on the viability of human glioblastoma U-87 MG cells and normal human NHA astrocytes, a WST-1 assay was conducted (Figure 1). Concentrations of the isoflavone spanned from 1 to 100 μM, and cell exposure to it lasted 48 h. A significant decrease in the viability of U-87 MG cells was observed at each NBIF concentration used, ranging from 1 μM (by ca. 12% of control) to 100 μM (by ca. 42% of control). In NHA cells, a significant viability reduction was noted at the two highest concentrations, i.e., 75 μM (ca. 11% of control) and 100 μM (ca. 28% of control). The viability of astrocytes and glioblastoma cells for the highest tested concentration was estimated at 72.17% (±6.28) and 58.23% (±7.69), respectively. The IC_75_ values of NBIF were calculated in GraphPad Prism software and were estimated at 36.6 μM in U-87 MG cells and 96.3 μM in NHA cells.

**Figure 2:**
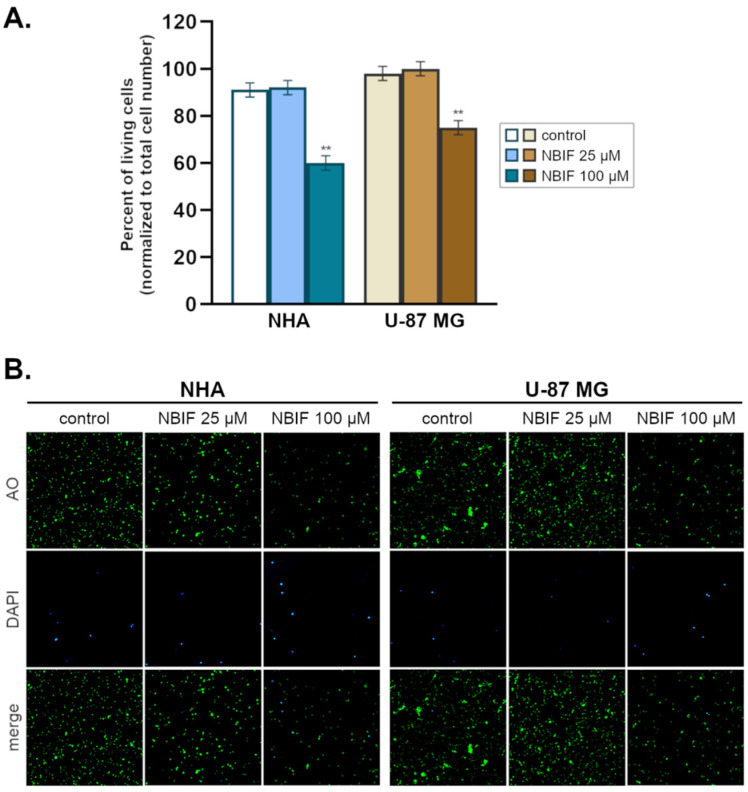
The growth inhibitory effect of neobavaisoflavone (NBIF) on NHA human astrocytes and U-87 MG glioblastoma cells. Groups were treated with NBIF (25 μM and 100 μM). (**A**) The number of living cells was determined by cell count assay using acridine orange (AO) and DAPI staining, represented as percent of all cells (living and dead) in the control. Mean values ± SD of three independent experiments are presented. ** *p* < 0.001 vs. control. (**B**) Representative images were acquired with the NC-3000 fluorescence image cytometer for the controls, and samples treated with NBIF.

Additionally, to evaluate the influence of NBIF on the proliferation of U-87 MG and NHA cells, a counting assay was performed (Figure 2A,B). For this purpose, two concentrations of NBIF were selected (25 μM and 100 μM) and incubated with cells for 48 h. A significant reduction in the number of the living cells (Figure 2A) was observed at the highest concentration (100 μM) in both NHA and U-87 MG cells, by ca. 31% and 23% (compared to the controls containing the total number of cells, as 100%), respectively. The percentage of living cells in the groups treated with 100 μM NBIF compared to the controls was estimated at approx. 60% in NHA astrocytes and 75% in U-87 MG cells. The number of the dead cells (stained with DAPI) was minute in every group (Figure 2B).

**Figure 3:**
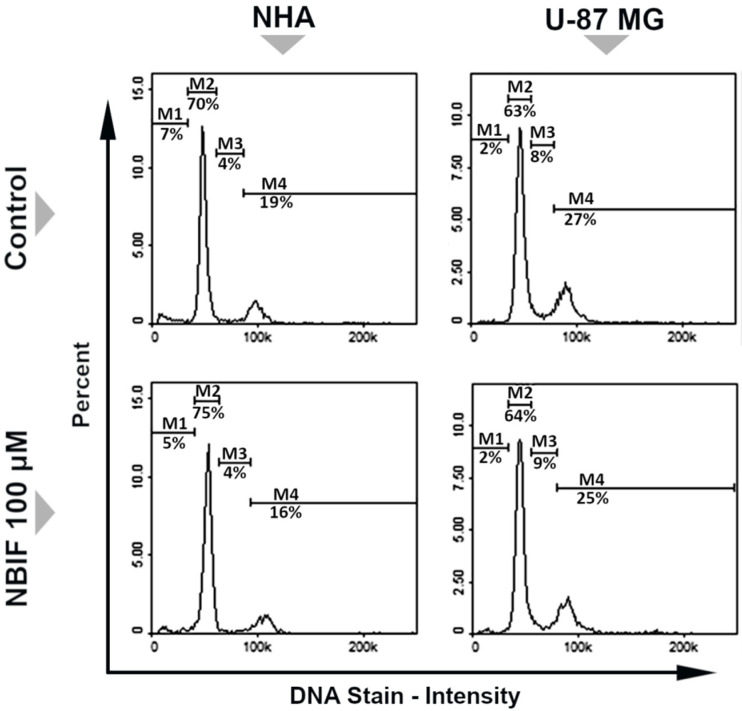
Cell cycle distribution of NHA and U-87 MG cells treated with neobavaisoflavone (NBIF) at a concentration of 100 µM, incubated for 48 h. The analysis was performed using a fluorescence image cytometer after staining cells with DAPI. The presented histograms are representative of three independent experiments with similar results. Marked cell cycle phases: M1–sub-G_1_, M2–G_1_/G_0_, M3–S, M4–G_2_/M.

The analysis of the cell cycle profiles of NHA and U-87 MG cells after the exposure to NBIF was performed using a fluorescence image cytometer (Figure 3). The subpopulations of cells were distributed throughout four phases of the cell cycle: G_1_/G_0_ phase, where one set of chromosomes per cell persists; S phase, in which DNA synthesis takes place; G_2_/M phase, where two sets of paired chromosomes per cell prior to the division are present; and sub-G_1_ phase consisting of cells containing less than one DNA equivalent (fragmented DNA). As shown in Figure 3, NBIF caused only slight changes in the cell cycle distribution. In NHA cells, the percentage of G_2_/M fraction was decreased from 19% to 16%, and in U-87 MG from 27% to 25%. Additionally, an increase in G_1_/G_0_ fraction of NHA cells treated with NBIF from 70% to 75% was noted.

### 2.2. NBIF Causes Reduction in the Level of Cellular GSH in U-87 MG Cells

**Figure 4:**
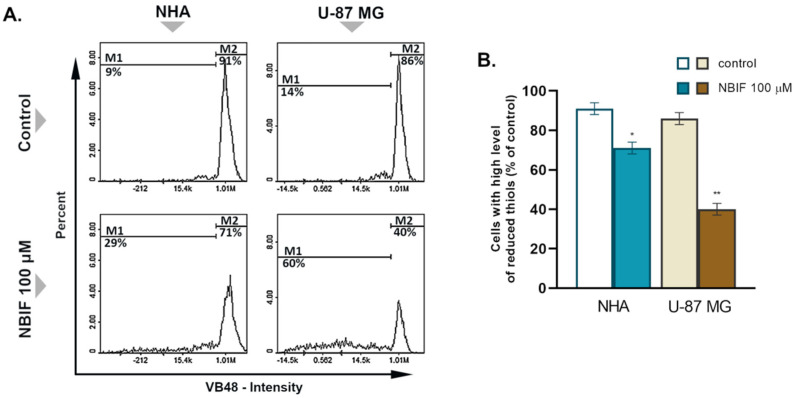
The impact of neobavaisoflavone (NBIF) on cellular GSH level in NHA human astrocytes and U-87 MG glioblastoma cells. (**A**) Histograms demonstrate a representative distribution of the cell populations for each tested group. Marker M1 represents the subpopulation of unhealthy cells with decreased levels of reduced thiols. Marker M2 represents healthy cells with a high level of reduced thiols. (**B**) Mean values ± SD of the percentage of cells with a high level of reduced thiols (bar graph) from three independent experiments in at least triplicate; statistically significant differences are designed as * *p* < 0.05, ** *p* < 0.001 vs. corresponding control using unpaired *t*-test.

Glutathione (GSH) is the major intracellular thiol, the reduced form of which persists in non-stress conditions, thus reflecting cell vitality and homeostasis [32]. To evaluate the impact of NBIF on the level of the GSH, cytometric analysis using a thiol-group-specific fluorescent dye, VitaBright-48™ (VB48), was performed. Since the cell count assay showed the effect only at the highest concentration, in this experiment, cells were treated with NBIF at 100 μM for 48 h. The results revealed a change in the GSH oxidation status in both NHA and U-87 MG cells (Figure 4A). NBIF caused a significant reduction in the subpopulation of cells with a high level of reduced thiols by 20% in NHA astrocytes and 40% in U-87 MG cells, as compared to the controls (Figure 4B).

### 2.3. The NBIF Effect on the Activity of Irinotecan, Etoposide, Doxorubicin, and Temozolomide on the Cell Viability

**Figure 5:**
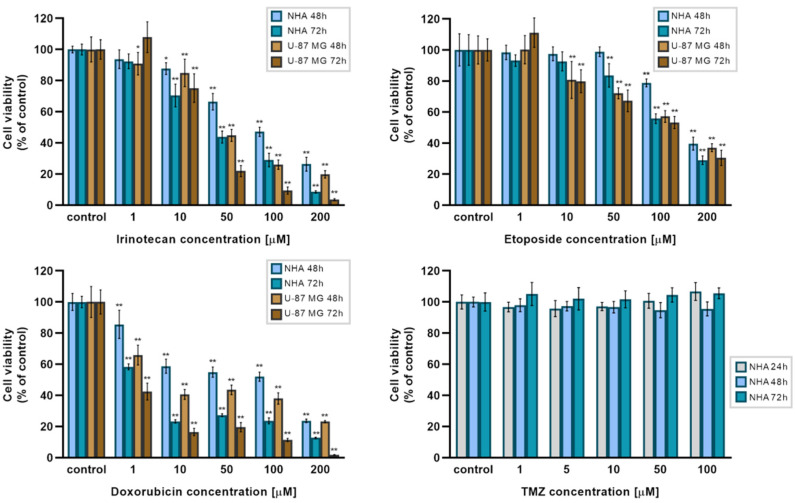
The effect of irinotecan, etoposide, doxorubicin, and temozolomide (TMZ) on NHA human astrocytes and U-87 MG glioblastoma cells. Cells were treated with increasing concentrations of irinotecan, etoposide, and doxorubicin (1 μM–200 μM) for 48 h and 72 h. TMZ (1 μM–100 μM) was added to NHA cells and incubated for 24 h, 48 h, and 72 h (lower-right panel). Data are presented as percent of the controls. * *p* < 0.05, ** *p* < 0.001.

Several studies have shown that isoflavones influence the effects of some chemotherapeutic agents, including topoisomerase inhibitors [33,34,35,36,37,38,39]. A WST-1 assay was performed to evaluate if NBIF influences the activity of irinotecan, etoposide, and doxorubicin. First, in the preliminary part of the experiment, we examined the effect of irinotecan, etoposide, and doxorubicin at concentrations ranging from 1 μM to 200 μM on the cell viability in NHA and U-87 MG cells in 48 h and 72 h (Figure 5). In cancer cells treated with irinotecan and etoposide, significant differences (*p* < 0.001) compared to the controls were noted at concentrations starting from 10 μM. Similar results were observed in NHA cells treated with irinotecan, but in astrocytes incubated with etoposide, a significant reduction in cell viability was found at 50 μM and higher concentrations. In groups treated with doxorubicin, significant differences were spotted in every used concentration. Additionally, to inspect the effect of TMZ on NHA cells, we performed a WST-1 assay in groups treated with this agent at concentrations of 1 μM to 100 μM for 24 h, 48 h, and 72 h (lower-right panel), as according to Respondek et al. [7] TMZ had no effect on U-87 MG cells in these concentrations. We retrieved similar results in astrocytes. The IC_50_ values for irinotecan, etoposide, and doxorubicin for 48 h and 72 h exposure were calculated in GraphPad Prism software and are presented in Table 1.

**Figure 6:**
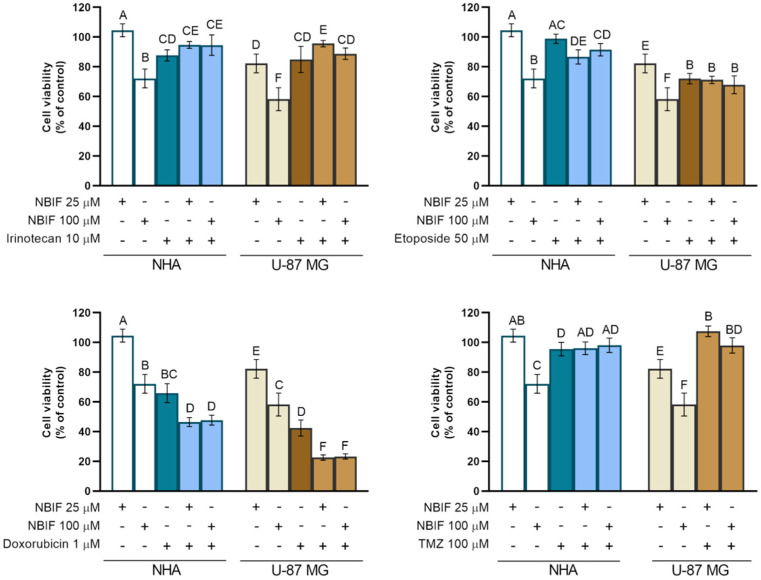
Comparison of the changes in the cell viability caused by NBIF alone (25 μM and 100 μM), drug alone (irinotecan 10 μM, etoposide 50 μM, doxorubicin 1 μM, or temozolomide (TMZ) 100 μM), and drug/NBIF mixture. Data are presented as percent of the controls, normalized to 100%. Values not sharing a common superscript differ significantly at *p* < 0.05.

For the next part of the experiment, concentrations of irinotecan, etoposide, and doxorubicin were selected (10 μM, 50 μM, and 1 μM, respectively) on the basis of the results obtained in the previous part. These were the lowest drug concentrations in groups incubated for 48 h, at which the statistically significant difference in the viability between NHA and U-87 MG cells had been present. We assumed that the viability of normal cells for the combined treatment should be as high as possible. For irinotecan, the lower concentration was chosen due to the large decrease in viability of normal cells at 50 μM. In the TMZ case, the highest concentration from the previous part of the study was used. To examine the effect of NBIF on the activity of these chemotherapeutics, we treated NHA and U-87 MG cells with mixtures containing irinotecan (10 μM), etoposide (50 μM), doxorubicin (1 μM), or TMZ (100 μM) combined with the isoflavone at 25 μM and 100 μM for 48 h and assessed the cell viability via the WST-1 test (Figure 6). No favorable effects were observed in groups incubated with NBIF combined with irinotecan, etoposide, or TMZ. Moreover, in U-87 MG cells treated with the mixture of NBIF (25 μM) and irinotecan or TMZ, an increase in the cell viability was noticed, as compared to drugs alone. Another unbeneficial effect was found in NHA astrocytes treated with the combination of NBIF (25 μM) and etoposide, where a slight reduction in the viability was observed. However, we found significant differences in cells treated with NBIF-doxorubicin mixture by ca. 19% in NHA cells and ca. 20% in U-87 MG cells, compared to groups treated with doxorubicin alone. The cell viability in these groups was estimated at approx. 47% and 23%, respectively.

## 3. Discussion

Treatment of GB is an immense challenge for modern medicine. Current regimens are usually unsuccessful and do not sufficiently improve survival; thus, new therapies need to be investigated [5,6]. Among the potential drug candidates are anti-neoplastic agents, such as topoisomerase inhibitors [11,12,13,14,15,16]. On the other hand, there are natural substances, such as isoflavones, that have proven to be effective against various cancer types [31]. There are also reports about their beneficial interactions with topoisomerase inhibitors [33,34,35,36,37,38,39]. In this paper, we investigated the effect of NBIF on the topoisomerase inhibitors’ activity, i.e., irinotecan, etoposide, and doxorubicin, on GB cells for the first time.

In several studies, NBIF has proven to decrease cell viability in cancer cells [27,28,29]. This effect was demonstrated by Kim et al. [29] in U-373 MG glioma cells. We found similar results in our study, where the reduction was observed at every used concentration in U-87 MG cells from 1 μM to 100 μM (Figure 1). Furthermore, herein we provide a comparison of the NBIF effect to normal cells, which are NHA astrocytes. In those cells, a decrease in the viability was not noticed at the low concentrations of NBIF (0 μM–50 μM). This was assumed as a sign of no cytotoxicity in normal cells, which is desirable for potential anti-cancer agents. We also demonstrated that NBIF at 100 μM inhibits cell growth by measuring the number of the U-87 MG and NHA cells treated with the isoflavone (Figure 2). However, we did not notice any significant alterations in the distribution of the cell cycle caused by NBIF (Figure 3). This might suggest that it inhibits cell growth in a cell cycle-independent manner. Together with the fact that we did not observe signs of cell death caused by NBIF, we suppose that this compound has anti-proliferative properties. This is in line with NBIF being considered an inhibitor of DNA polymerase and topoisomerase II, enzymes crucial for cell proliferation [40]. 

GSH is necessary in numerous cellular processes, such as proliferation; thus, its imbalance may lead to the inhibition of cell growth [41]. In cancer cells, the GSH level is typically elevated in order to buffer the augmented oxidative stress caused by increased metabolism sufficiently. It is also involved in the detoxification of xenobiotics. Depletion of GSH, therefore, results in oxidative damage to DNA and cell organelles. Moreover, excessive levels of GSSG contribute to this, as it acts as a pro-oxidant [42]. In fact, flavonoids are able to induce a reduction in the intracellular level of GSH [43,44]. Although these compounds are characterized by their anti-oxidant properties, depending on the conditions, they are able to inhibit anti-oxidant defense system of the cell [45]. This might be due to the modulation of transcriptional factors involved in GSH recycling, e.g., nuclear factor erythroid 2-related factor 2 (Nrf2), as polyphenols have been proved to act in this manner [46,47]. Herein, we report for the first time that NBIF triggers GSH depletion in GB cells (Figure 4A,B). In our experiment, this compound at a concentration of 100 μM caused an over 2-fold decrease in the subpopulation with a high level of GSH in U-87 MG cells, and 1.28-fold in NHA astrocytes. 

Several isoflavones have been found to enhance the activity of anti-cancer drugs [28,29,30,31,32,33,34]. Since GSH depletion is considered a mechanism to sensitize cancer cells to chemotherapeutical agents [36,37], we investigated if NBIF-induced GSH reduction influences the activity of irinotecan, etoposide, doxorubicin currently used in GB therapy TMZ (Figure 5 and Figure 6). Among the drugs used, in groups treated with irinotecan, etoposide, and TMZ we did not notice any significant favorable effects of the combination with NBIF on the cell viability (Figure 6). Interestingly, NBIF as an additional anti-proliferative agent did not affect the reduction in the cell viability in these groups when compared to cells treated only with drug. It seems like the only agent that caused the effect was the chemotherapeutic. However, we discovered a potentializing action in cells treated with the NBIF doxorubicin mixture. In U-87 MG cells, the reduction in the viability was almost 2-fold compared to cells treated with the drug alone and greater than in NHA astrocytes. In the literature, it has been reported that some isoflavones exhibit sensitizing action when combined with doxorubicin. One of the studies concerning this subject was by Xue et al. [30], where genistein sensitized drug-resistant human breast cancer cells to doxorubicin. Another studied isoflavone, biochanin A, has been demonstrated to have similar properties. It has been shown to inhibit proliferation in osteosarcoma cells [33] and reverse drug resistance in colon cancer cells [34]. There is also a study conducted on glioma cells, i.e., on U-87 MG cells, by Liu et al. [31], in which formononetin, a soy isoflavone, was proven to enhance the effect of doxorubicin. Our findings are consistent with these experiments, and in comparison, to the results on formononetin treated U-87 MG cells, NBIF was shown to be more effective alone at the same concentrations, but when combined with doxorubicin, reduces the cell viability in a similar manner. 

Our work is a first step in exploring NBIF antiglioblastoma properties concerning interactions with topoisomerase inhibitors. We demonstrated that this isoflavone potentiates the effect of doxorubicin. Thus, our findings are a strong basis for future research, oriented to improving the knowledge about mechanisms underlaying our findings, especially molecular processes.

## 4. Materials and Methods

### 4.1. Materials

Human glioblastoma cells (U-87 MG), neobavaisoflavone (7-hydroxy-3-(4-hydroxy-3-(3-methyl-2-buten-1-yl)phenyl)-4H-1-benzopyran-4-one), penicillin G, and dimethyl sulfoxide (DMSO) were purchased from Sigma-Aldrich Inc. (St. Louis, MO, USA). Dulbecco’s modified Eagle’s medium (DMEM) and fetal bovine serum were retrieved from Cytogen (Zgierz, Poland). Gibco Human Astrocytes (NHA) and Gibco Astrocyte Medium (DMEM, N-2 Supplement, One Shot fetal bovine serum), Trypsin/EDTA solution were acquired from ThermoFisher Scientific (Waltham, MA, USA). NC Slides™ A8 and Via1-Cassettes™, as well as Solution 3 (1 μg/mL DAPI, 0.1% Triton X-100 in PBS) and Solution 5 (400 μg/mL VitaBright™ 48, 500 μg/mL propidium iodide (PI), 1.2 μg/mL acridine orange in DMSO) were obtained from ChemoMetec (Lillerød, Denmark). Neomycin sulfate was purchased from Amara (Kraków, Poland). Cell Proliferation Reagent WST-1 was purchased from Roche (Mannheim, Germany). In the study, the following drugs were used: irinotecan (Irinotecan Accord, Accord, Ahmedabad, India), etoposide (Etoposid-Ebewe, Ebewe Pharma, Ahmedabad, India), doxorubicin (Doxorubicin Accord, Accord, Ahmedabad, India). The rest of the chemicals were acquired from POCH S.A. (Gliwice, Poland).

### 4.2. Cell Culture

U-87 MG cells were cultured in DMEM medium supplemented with fetal bovine serum, and NHA cells were incubated in Gibco Astrocyte Medium, consisting of DMEM, N-2 Supplement, One Shot fetal bovine serum. All used media were supplemented with penicillin G (10,000 U/mL), neomycin (10 μg/mL), and amphotericin B (0.25 mg/mL). U-87 MG and NHA cultures were maintained at 37 °C in humidified 5% CO_2_ atmosphere. The experiments were performed at passages 8–12 of NHA cells.

### 4.3. Cell Viability Assay

A WST-1 (4-[3-(4-iodophenyl)-2-(4-nitrophenyl)-2H-5-tetrazolio]-1,3-benzene disulfonate) colorimetric assay was used to estimate the number of viable cells. The principle of this analysis is based on the action of mitochondrial dehydrogenases catalyzing WST-1 reduction, which correlates with the number of viable cells. NHA and U-87 MG cells were seeded in supplemented culture media (Gibco Astrocyte Medium for NHA cells and DMEM for U-87 MG) in 96-well microplates at 2500 cells per well to determine cell viability in the experiment. Cells were incubated in humidified 5% CO_2_ atmosphere at 37 °C for 48 h (NHA) and 24 h (U-87 MG). Then, media were removed and cell cultures were treated with NBIF (1 μM, 5 μM, 10 μM, 25 μM, 50 μM, 75 μM, 100 μM), irinotecan (1 μM, 10 μM, 50 μM, 100 μM, 200 μM), etoposide (1 μM, 10 μM, 50 μM, 100 μM, 200 μM), doxorubicin (1 μM, 10 μM, 50 μM, 100 μM, 200 μM), temozolomide (1 μM, 5 μM, 10 μM, 50 μM, 100 μM), and NBIF (25 μM, 100 μM) combined with: irinotecan (10 μM), etoposide (50 μM), doxorubicin (1 μM), and temozolomide (100 μM). After 48 h of incubation, 10 μL of WST-1 reagent was added to each well containing 100 μL of media. Following 3 h of incubation, the absorbance of the samples was measured at a wavelength of 440 nm and 650 nm as a reference wavelength using an Infinite 200 PRO (TECAN, Männedorf, Switzerland) microplate reader. The controls were normalized to 100% for each assay, and treatments were demonstrated as the percentage of the controls.

### 4.4. Cell Count Assay

NucleoCounter NC-3000 fluorescence image cytometer controlled by NucleoView NC-3000 Software (Chemometec, Denmark) was used to determine the total number of NBIF-treated and untreated (controls, 0.1% DMSO) NHA and U-87 MG cells. In brief, cells were harvested by exposure to trypsin/EDTA solution and loaded into Via1 Cassette (Chemometec, Denmark), which contained DAPI (4′,6′-diamidino-2-phenylindole) and acridine orange (AO).

### 4.5. Cell Vitality Assay—Assessment of the Level of Cellular Reduced Glutathione (GSH)

In order to evaluate the intracellular level of the reduced GSH, NHA, and U-87 MG, cells were seeded in T-75 flasks at a density of 0.8 × 10^6^ per flask and after 48 h treated with NBIF (100 μmol/L) for 48 h. After the incubation, cells were collected by trypsinization and counted. One volume (10 μL) of Solution 5 containing acridine orange that stains all cells, PI that stains dead cells only, and VitaBright-48™ (VB48), which stains viable cells with an intensity depending on the level of thiols, was added to 19 volumes (190 μL) of medium containing 1 × 10^6^ suspended cells. The stained-cell suspensions were put into NC-Slides™ A8 and analyzed by NucleoCounter NC 3000 fluorescence image cytometer using the Vitality assay protocol.

### 4.6. Fixed Cell Cycle-DAPI Assay

The analysis of the cell cycle was assessed using NucleoCounter NC 3000 fluorescence image cytometer. Cells were seeded in T-75 flasks (at a density of 0.8 × 106 per flask). After 48 h, they were treated with NBIF (100 µmol/L) for 48 h, then harvested by trypsinization and counted. One million cells were suspended in 0.5 mL PBS and fixed with 4.5 mL of 70% cold ethanol for at least 2 h. Then, the ethanol was removed, cells were washed with PBS, and centrifuged for 5 min at 500× *g*. To the pellets of cells, 0.5 mL of Solution 3 (containing DAPI and cell membrane-disrupting Triton X-100) was added. After 5 min of incubation at 37 °C, stained cells were loaded into NC-Slide A8™ and analyzed by NucleoCounter NC 3000 fluorescence image cytometer using Fixed Cell Cycle-DAPI assay protocol.

### 4.7. Statistical Analysis

In all experiments, the mean values of at least three separate experiments performed in triplicate ± SD were calculated. Differences between groups were analyzed in GraphPad Prism 8.0 (GraphPad Software, San Diego, CA, USA) program by unpaired t-test or one-way ANOVA followed by post-hoc Dunnett’s or Tukey’s test, as appropriate. A *p*-value lower than 0.05 was considered indicative of a statistically significant difference.

## 5. Conclusions

Taken together, our results shed new light on the anti-cancer properties of NBIF. Herein, we report for the first time that this compound at a concentration of 100 μM causes GSH depletion in U-87 MG cells, exhibits anti-proliferative effect against them, and sensitizes them to doxorubicin. Therefore, further studies of the molecular basis underlying the NBIF action need to be carried out.

## Figures and Tables

**Table 1:** The IC_50_ values of irinotecan, etoposide and doxorubicin with the duration of the exposure.

	Irinotecan (μM)		Etoposide (μM)		Doxorubicin (μM)	
	48 h	72 h	48 h	72 h	48 h	72 h
NHA	85.9	30.3	168.9	117.7	45.9	1.6
U-87 MG	42.4	21.5	126.6	97.2	7.6	0.4

## Data Availability

The data that support the findings of this study are available from the corresponding author upon reasonable request.

## Sample Availability

Samples of the compounds are not available from the authors.
